# Phytosterol esters attenuate hepatic steatosis in rats with non-alcoholic fatty liver disease rats fed a high-fat diet

**DOI:** 10.1038/srep41604

**Published:** 2017-02-07

**Authors:** Lihua Song, Dan Qu, Qing Zhang, Jing jiang, Haiyue Zhou, Rui Jiang, Yating Li, Yao Zhang, Hongli Yan

**Affiliations:** 1 Research Center for Food Safety and Nutrition, Key Lab of Urban Agriculture (South), Bor S. Luh Food Safety Research Center, School of Agriculture & Biology, Shanghai Jiao Tong University, Shanghai 200240, China; 2 Nutrition Department, Shanghai Seventh People’s Hospital, Shanghai 200137, China; 3 Department of Laboratory Medicine, Changhai Hosipital, Second Millitary Medical University, Shanghai 200433, China

## Abstract

Given the adverse effects of drugs used for NAFLD treatment, identifying novel and effective natural compound to prevent NAFLD is urgently needed. In the present study, the effects of phytosterol esters (PSEs) on NAFLD were explored. Adult SD rats were randomized into five groups: normal chow diet (NC), high-fat diet (HF), low-, medium- and high-dose PSE treatment plus high-fat diet groups (PSEL, PSEM, and PSEH). Our results showed that the levels of LDL-C in the PSEL group and hepatic TG, TC, and FFA in the three PSEs groups were significantly decreased. Notably, the uric acid (UA) level was significantly decreased by PSEs intervention. The hepatic inflammatory stress was ameliorated via the inhibition of the cytokines, including TGF-β, IL-6, IL-10 and CRP in the PSEs intervention groups. Further, the oxidative status was improved by PSE treatment through adjusting the enzyme activity (SOD and XOD) and decreasing the MDA level. These beneficial effects of PSE may have been partly due to its regulation on the expression of TGF-β1, TGF-β2, TNF-α, UCP-2, PPAR-α and PPAR-γ in hepatic tissue at both mRNA and protein level. The results of this study suggest that PSEs may be used as therapeutic agents for the prevention and progression of NAFLD and that hyperuricemia is induced by high-fat diet consumption.

In recent years, the prevalence of non-alcoholic fatty liver disease (NAFLD) in the general population has been rapidly increasing because of lifestyle changes, e.g., increased consumption of high-fat diets and lack of exercise, which also are the main causes of chronic diseases such as obesity, dyslipidemia and hyperglycemia. Although most patients with NAFLD have few or no symptoms, it is becoming an increasingly frequent cause of chronic liver damage because of its potential to progress to steatohepatitis, fibrosis, cirrhosis and even hepatocellular carcinoma. More importantly, NAFLD is now recognized as an important health concern worldwide since it is also an independent risk factor for type 2 diabetes and cardiovascular disease^1^,^2^.

The pathogenesis of NAFLD is highly complex and multifactorial, and a “two-hit” hypothesis has been proposed to explain it ref. 3. According to this hypothesis, the first hit involves accumulation of fatty acids (FAs)/triglycerides (TGs) in the liver and the development of insulin resistance (IR), whereas the second hit involves oxidative stress and inflammation, which ultimately cause liver damage^4^. Hepatic lipid accumulation plays a pivotal role in the pathogenesis and progression of NAFLD. In previous studies, the dysregulation of cholesterol metabolism has received much less attention than the involvement of FAs and TGs in this disease^5^. However, the most recent findings of interest have shown that cholesterol intake is significantly increased in non-obese NAFLD patients compared with obese NAFLD patients^6^. Excess cholesterol intake in itself may strongly promote the development of steatosis because hepatic cholesterol accumulation can lead to activation of the liver X receptor α-sterol regulatory element-binding protein-1c (LXRα-SREBP-1c) pathway, which is closely related to the development of NAFLD^7^. It has also been reported that HMG-CoA reductase inhibitors (e.g., statins) and cholesterol absorption inhibitors reduce serum alanine aminotransferase (ALT) levels in NAFLD patients^8^. Therefore, the regulation of cholesterol metabolism under high-fat diet condition may represent a reliable therapeutic strategy for NAFLD.

A large number of studies have shown that phytosterols regulate the blood lipid profile and lower total cholesterol (TC) and low-density lipoprotein cholesterol (LDL-C) levels. Food products containing these plant compounds are widely used as therapeutic dietary options for reducing hypercholesterolemia and the atherosclerotic risk^9^,^10^,^11^. Importantly, a series of studies have demonstrated that phytosterols exhibit antioxidant potential. Carange J *et al*.^12^ proved that 24-epibrassinolide, a phytosterol from the brassinosteroid family, protects neuronal cells from MPP-induced oxidative stress and apoptosis. In addition, Gupta R *et al*.^13^ have demonstrated that β-sitosterol has promising antidiabetic and antioxidant effects in streptozotocin-induced experimental hyperglycemia.

Considering their cholesterol-lowering, antioxidant and hypoglycemic effects, we hypothesized that phytosterols would attenuate events leading to NAFLD. To prove this hypothesis, the present study was designed to evaluate the preventive effects of phytosterols on NAFLD using an animal model fed a high-fat diet. Different doses of phytosterol esters (PSEs) were used to fortify skim milk, which was ingested by the experimental animals daily. The lipid profile, liver and kidney function, hepatic lipid content and histopathological changes were observed. In addition, the influences of PSEs on cytokines and oxidative markers were examined and the genes related to lipid metabolism and inflammation were analyzed to further elucidate the effects of PSEs on NAFLD.

## Results

### Intake of PSEs did not influence growth

The weights of the SD rats in the different group did not significantly differ (*P* > 0.05) at the beginning of the experiment (t_0_). The average food intake of the rats in the NC group was slightly higher than that of the rats in all of the high-fat diet groups, but no significant differences were observed in food consumption (Supplementary Fig. S1). At the end of the experiment (t_12_), the weights of the high-fat diet-fed rats were slightly higher than those of the rats in the NC group, but no significant differences were observed among all the five groups, suggesting that PSE intake did not influence rat growth (Supplementary Table S2).

### Effects of PSEs on serum lipids and fasting blood glucose levels in rats

**Figure 1:**
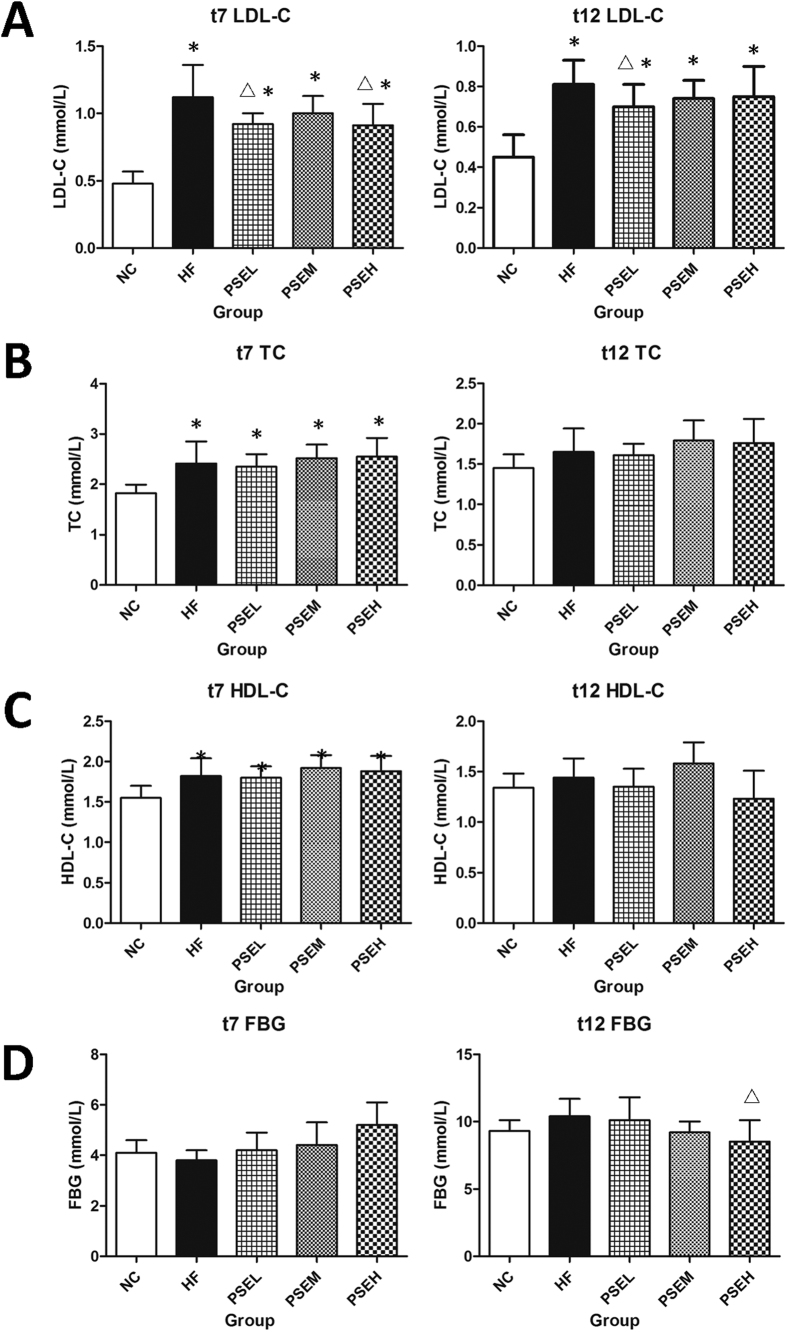
Effects of PSEs on serum lipid and glucose levels in rats at t_7_ and t_12_. (**A**) LDL-C; (**B**) TC; (**C**) HDL-C; and (**D**) FBG. NC: Normal control group (n = 6); HF: high-fat diet group (n = 12); PSEL: low-dose PSEs treatment group (0.05 g/100 g·BW, n = 12); PSEM: medium-dose PSEs treatment group (0.10 g/100 g·BW, n = 12); and PSEH: high-dose PSEs treatment group (0.15 g/100 g·BW, n = 12). t_7_ and t_12_ indicate data measured at the 7^th^ week of the experiment and at the end of the experiment, respectively. The data are expressed as the mean ± standard deviation. *P < 0.05 *vs.* NC group; ^Δ^*P* < 0.05 *vs.* HF group.

The effects of PSEs on the serum lipid and fasting blood glucose levels in the rats are shown in Fig. 1A–D. At weeks 7 (t_7_) and 12 (t_12_), the rat serum LDL-C levels in the HF group and three PSEs intervention groups were significantly higher than those in the NC group (*P* < 0.05), while the LDL-C levels in the PSEL group were decreased by 18% and 14%, respectively, compared with those in the HF group (Fig. 1A). Increasing PSEs doses did not cause a further reduction in LDL-C. In addition, the fasting blood glucose (FBG) level in the high-dose PSEH group was significantly decreased after 12 weeks of PSEs intervention (Fig. 1D) (*P* < 0.05).

### Effects of PSEs on rat hepatic fat content

**Figure 2:**
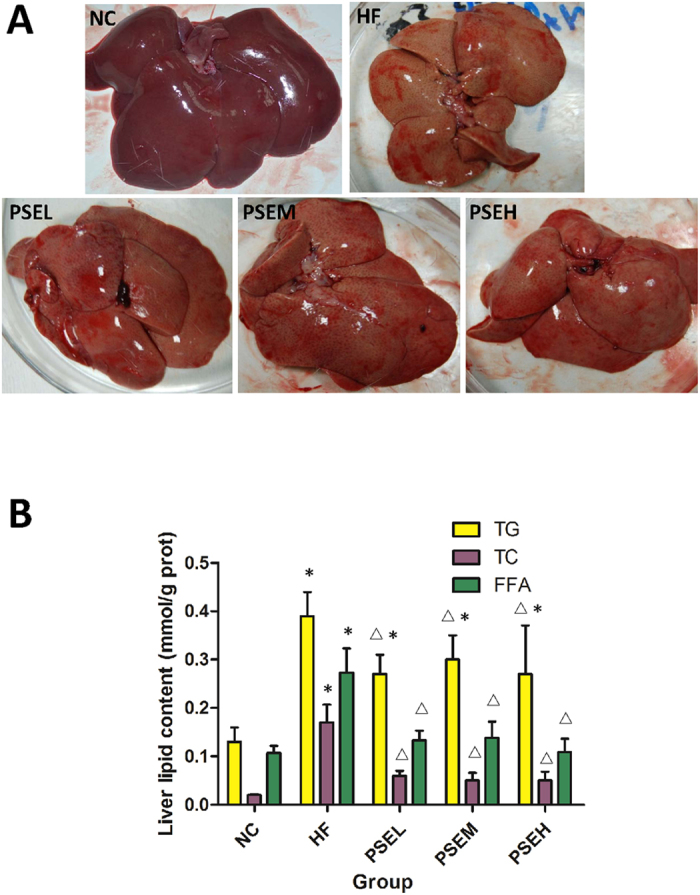
Effects of PSEs on hepatic fat content in rats. (**A**) Comparison of liver appearance among the NC, HF and three PSEs treatment groups. (**B**) The hepatic TG, TC and FFA concentrations in the livers of the rats in the different groups. The NC, HF, PSEL, PSEM and PSEH groups are described as Fig. 1. The liver anatomical appearance was greatly improved by PSEs intervention. The PSEs treatments at the three doses significantly decreased the hepatic TG, TC and FFA levels compared with those in the HF group. The data are expressed as the mean ± standard deviation. *P < 0.05 *vs.* NC; ^Δ^*P* < 0.05 *vs.* HF.

As shown in Fig. 2A, none of the rats in the NC group developed obvious pathological changes; the liver color was red-brown and the surface morphology was normal at the end of the experiment. Pathological abnormalities were observed in the HF group, including a yellow-brown liver color and lipid accumulation. However, following treatment with PSEs at the three doses, the liver color and surface morphology were greatly improved compared with the HF group.

Hepatic lipid content assay was performed to assess the effects of PSEs on lipid accumulation in the liver. Figure 2B shows that after 12 weeks of high-fat diet consumption, the liver lipid content was significantly higher in the HF group than in the NC group (*P* < 0.05), while the hepatic TG, TC, and FFA levels were significantly decreased by 31%, 65% and 51%, respectively, in the PSEL group (0.05 g/100 g BW) compared with the HF group (*P* < 0.05). Further increases in the PSEs dose did not lead to a more pronounced lipid-lowering effect.

### PSEs prevent steatosis induced by high-fat diet

**Figure 3:**
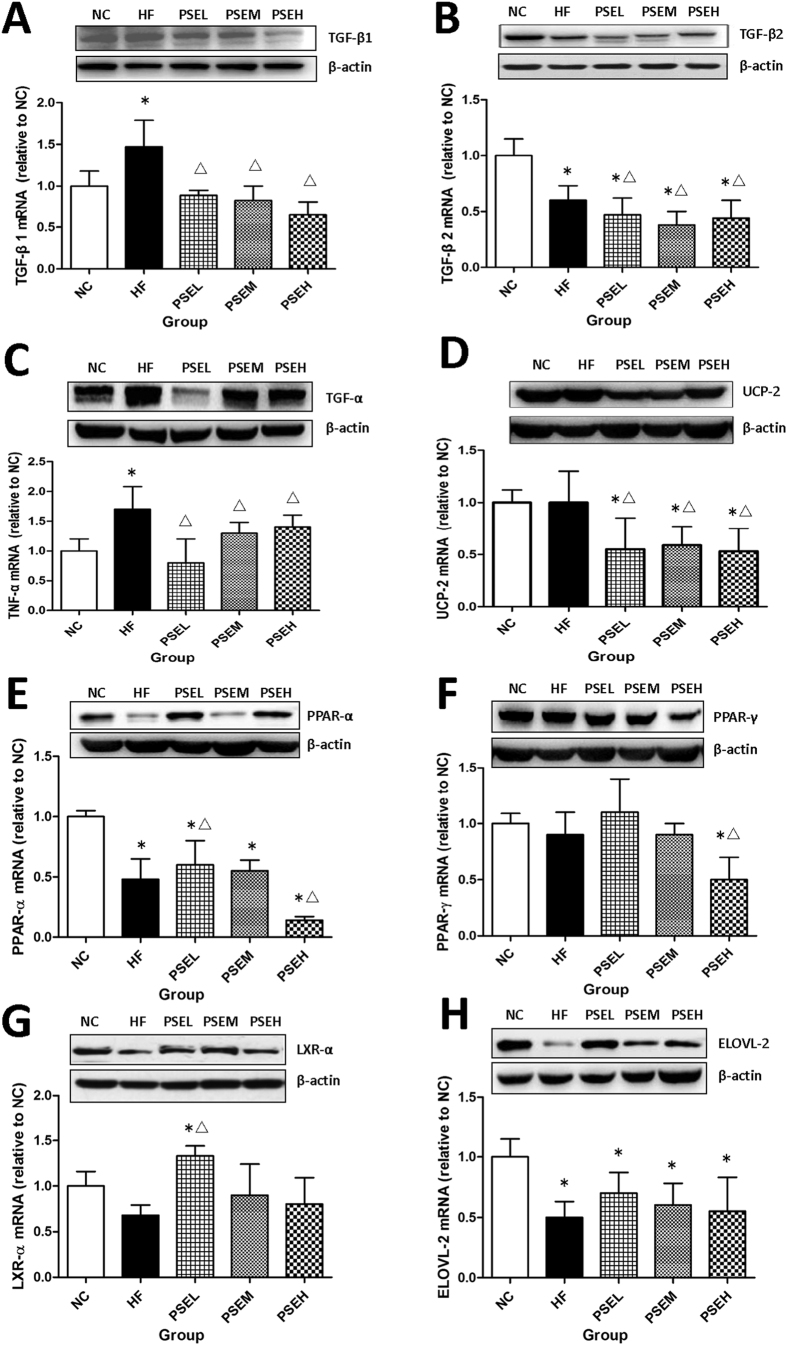
The liver pathologies in the different groups of NAFLD rats. (**A**) Hematoxylin and eosin (HE) staining (200×) and hepatic steatosis scores. (**B)** Oil Red O staining of liver tissues (200×). The NC, HF, PSEL, PSEM and PSEH groups are described in Fig. 1. The hepatic steatosis degree was decreased by PSEs intervention compared with that in the HF group.

Figure 3A shows the pathological results for the liver tissues from the rats in the various groups following hematoxylin and eosin (HE) staining, as well as the steatosis scores. The livers of the HF group rats showed significant steatosis with damaged lobular structures, absence of the hepatic sinusoid, enlarged liver cells, blurred boundaries between cells, damaged cell membranes, lipid droplets in the cytoplasm and pushing of the nucleus to the side, as well as the formation of lipid vacuoles by numerous lipid droplets. Compared with the hepatocytes from the rats in the HF group, those from the rats in the PSEs intervention groups did not have large lipid vacuoles, the lipid droplet diameter, number of lipid droplets, and area covered by the lipid droplets were significantly lower, the boundaries between hepatocytes were more obvious, and it was possible to identify lobular structures. The steatosis scores, evaluated by a pathologist, revealed that hepatic steatosis was more severe in the HF group than in the PSEs intervention groups (*P* < 0.05).

In addition, the liver tissues were cryo-sectioned and stained with Oil Red O, and the results revealed no fat deposition or scarce deposition in the liver tissues from the NC group, with small red areas, indicating the presence of few fat droplets in the liver cells in the liver tissue sections. In contrast, the liver tissues from the rats in the HF group accumulated an increased number of larger lipid droplets (indicated by the presence of red dots, representing fat) and presented with significant steatosis. The degree of steatosis was lower in all PSEs intervention groups than in the HF group, and it was particularly decreased in the PSEL group (Fig. 3B). These results strongly suggest that PSEs have certain preventive effects on high-fat diet-induced NAFLD.

### Effects of PSEs on rat liver function

Table 1 shows the results of the liver function tests used to evaluate the rats at different time points. At week 7 (t_7_), the ALT, alkaline phosphatase (ALP) and TP levels were significantly higher in the HF group and three PSE intervention groups than in the NC group (*P* < 0.01). However, the serum glutamic oxalacetic transaminase (AST) levels in the three PSEs intervention groups were decreased to varying degrees compared with those in the HF group. In particular, the AST activity was significantly lower in the PSEM group than in the HF group (*P* < 0.05).

At week 12 (t_12_), the serum ALT and ALP levels significantly increased (*P* < 0.05) by 31 and 24%, respectively, and the AST level increased by 11% in the HF group compared with the NC group. After PSEs intervention, the rat serum AST, ALT and ALP levels exhibited downward trends compared with those in the HF group. The AST, ALT, and ALP levels decreased by 10, 5, and 7%, respectively, in the PSEL group compared with the HF group. Further, the ALT level in the PSEH group and ALP level in the PSEM group were significantly lower than those in the HF group (*P* < 0.05).

### Effects of PSEs on rat renal function

At t_7_, the serum creatinine (CRE) level in the HF group was significantly higher than that in the NC group (*P* < 0.01). Compared with the HF group, the different PSEs intervention groups showed differing degrees of reduction in the CRE level, and the CRE level in the PSEM group was significantly lower than that in the HF group (Table 2).

At t_12_, the serum CRE and uric acid (UA) levels were significantly higher in the HF group than in the NC group (*P* < 0.01), with increases of 23 and 51%, respectively. In addition, the CRE and UA levels were slightly decreased in the PSE intervention groups. Further, the CRE and UA levels in the PSEL group decreased by 13 and 39%, respectively, approaching the levels observed in the NC group. Notably, the UA levels were lower than in the HF group following treatment with low, medium and high doses of PSEs and were similar to the levels in the NC group, suggesting that PSEs prevent abnormalities in purine metabolism induced by high-fat diet consumption.

### Effects of PSEs on serum inflammatory factors and high-sensitivity C-reactive protein (hs-CRP)

**Figure 4:**
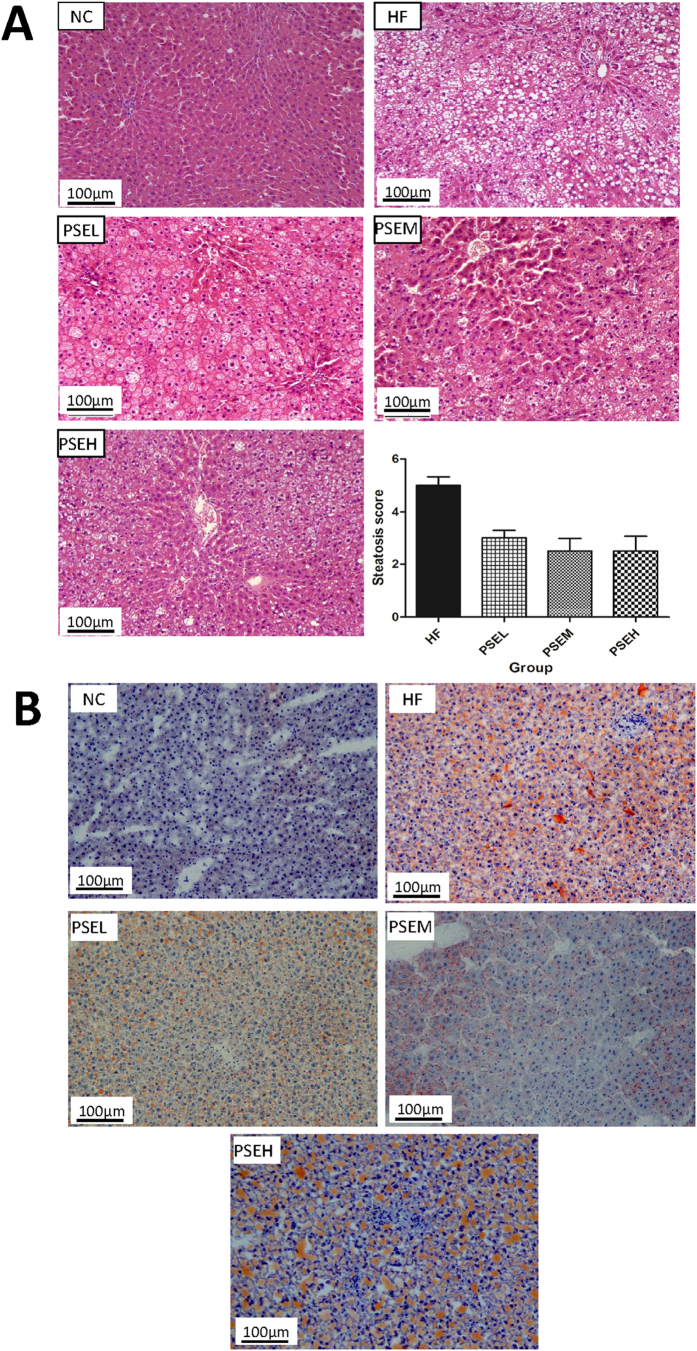
Effects of PSEs on serum inflammatory factors. The NC, HF, PSEL, PSEM and PSEH groups are described in Fig. 1. The serum CRP, TGF-β and IL-6 levels were significantly decreased in the three PSEs intervention groups, and the IL-10 level was significantly decreased in the PSEL and PSEM groups. The data are expressed as the mean ± standard deviation. *P < 0.05 *vs.* NC; ^Δ^*P* < 0.05 *vs.* HF.

The effects of PSEs on inflammatory factors and adipokines in rat serum are shown in Fig. 4(A–F). The serum interleukin (IL)-6, IL-10, tumor necrosis factor (TNF)-α and transforming growth factor (TGF)-β levels increased by 18, 32, 10 and 52%, respectively, in the HF group compared with the NC group (Fig. 4A–D), and the adiponectin concentration decreased by 32% (*P* < 0.05) (Fig. 4F).

The IL-6, IL-10 and TGF-β levels decreased significantly by 10, 26 and 35% (*P* < 0.05) (Fig. 4A,B,D), respectively, in the PSEL group compared with the HF group. Further, the leptin level in each PSEs intervention group was similar to that in the NC group and was slightly higher than that in the HF group (Fig. 4E), and the adiponectin levels were significantly lower in all PSEs intervention groups than in the NC group (*P* < 0.05), but they remained 7–10% higher than that in the HF group (Fig. 4F). The effects of high and medium PSEs doses on cytokine levels did not significantly differ from the effects of a low dose (Fig. 4A–F).

The level of hs-CRP, which is known to be a cardiovascular disease (CVD) risk factor, is elevated in NAFLD patients, particularly in those with NASH. Our present study result showed that this level was significantly higher in the HF group compared with the NC group, whereas it was successfully controlled in the three PSEs treatment groups (*P* < 0.05) and remained close to the level in the NC group, which further demonstrated the anti-inflammatory potential of PSEs (Fig. 4G).

### Effects of PSEs on anti-oxidant activity in rat liver tissues

**Figure 5:**
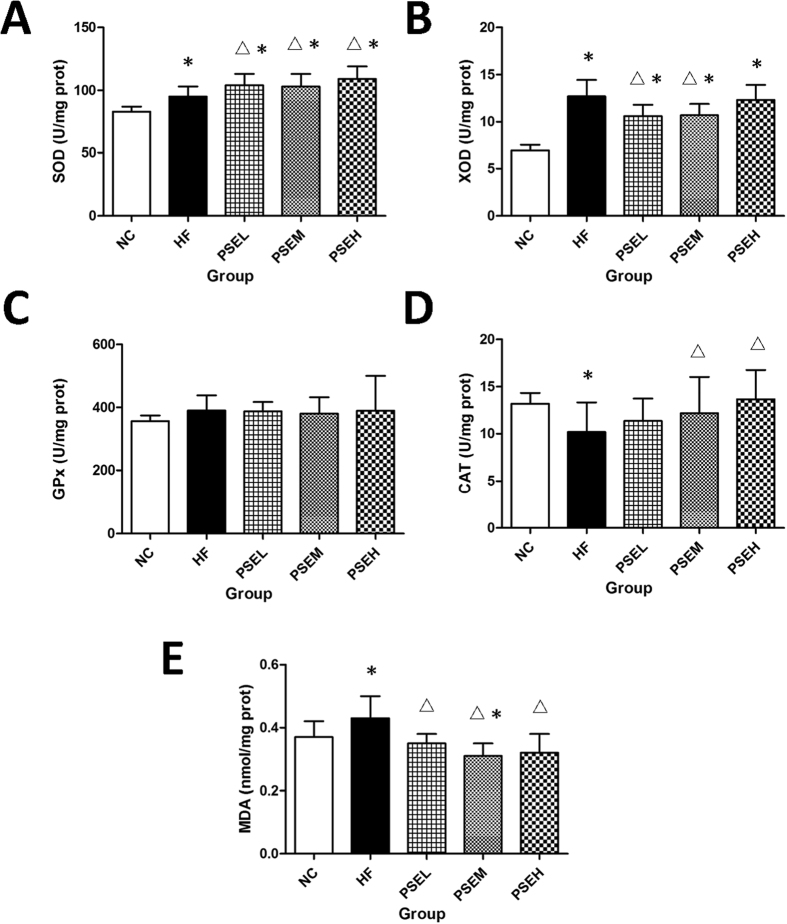
Effects of PSEs on oxidative stress markers in rat hepatic tissues. The NC, HF, PSEL, PSEM and PSEH groups are described in Fig. 1. SOD activity in the three PSEs treatment groups and CAT activity in the PSEM and PSEH groups were significantly increased compared with those in the HF group, XOD activity was significantly decreased in the PSEL and PSEM groups, and the MDA levels in all PSEs intervention groups were significantly decreased (*P* < 0.05). The data are expressed as the mean ± standard deviation. *P < 0.05 *vs.* NC; ^Δ^*P* < 0.05 *vs.* HF.

The effects of PSEs on *in vivo* anti-oxidant activity are shown in Fig. 5(A–E). The superoxide dismutase (SOD) and xanthine oxidase (XOD) activities significantly increased by 14 and 84%, respectively, and the malondialdehyde (MDA) level increased by 16% in the HF group compared with the NC group, and these differences were all significant (*P* < 0.05) (Fig. 5A,B,E). Compared with those in the HF group, SOD activity in the three PSEs treatment groups and catalase (CAT) activity in the PSEM and PSEH groups were significantly increased (*P* < 0.05), XOD activity was significantly decreased in the PSEL and PSEM groups (*P* < 0.05), and the MDA levels in all PSE intervention groups were significantly decreased (*P* < 0.05) (Fig. 5A,D,B,E). However, no significant alterations in glutathione peroxidase (GPx) activity were detected (Fig. 5C).

### Effects of PSEs on the expression of lipid metabolism- and inflammation-related genes

**Figure 6:**
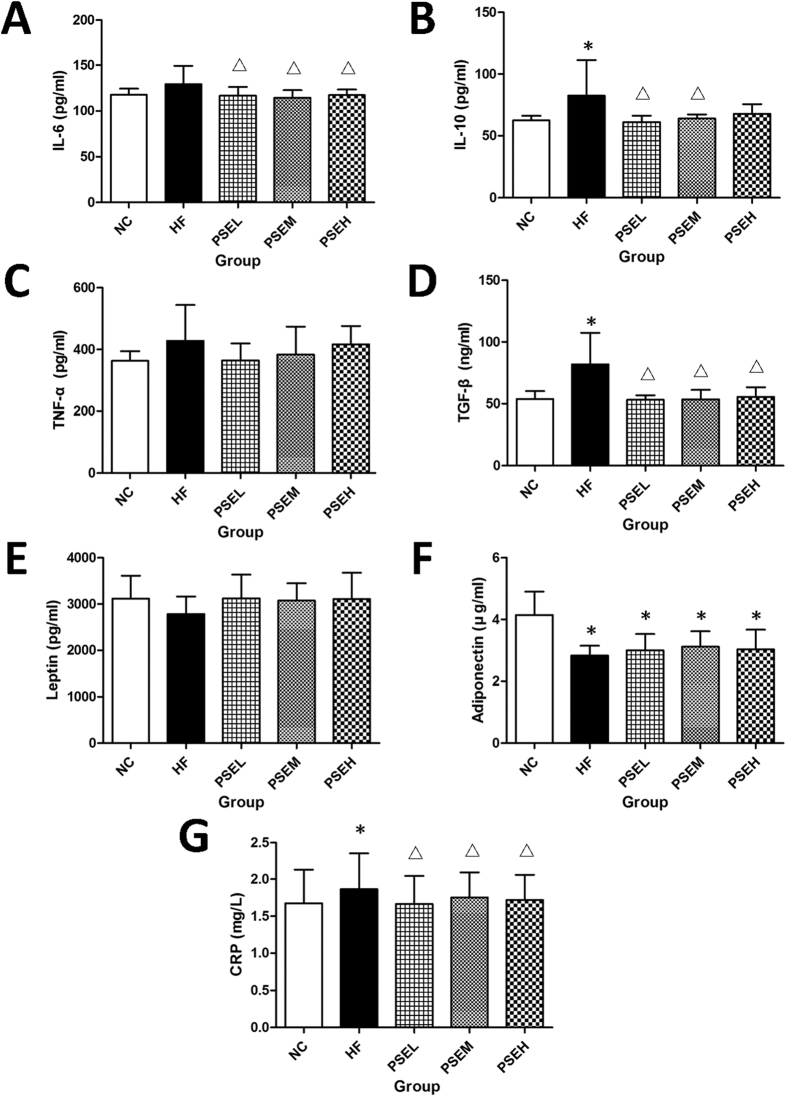
Gene expression in hepatic tissues in the different groups. The mRNA and protein expression levels in hepatic tissues in the HF and three PSEs intervention groups were compared with those in the NC group. The PSEs treatments at the three doses resulted in significant down-regulation of TGF-β1, TGF-β2, TNF-α, and UCP-2 expression and slight up-regulation of ELOVL-2 expression. The effects of PSEs on PPAR-α, PPAR-γ and LXR-α expression differed. The data are expressed as the mean ± standard deviation. ^Δ^*P* < 0.05 *vs.* HF.

To further investigate the molecular mechanisms of PSEs in alleviating the progression of NAFLD, the mRNA and protein levels of several lipid metabolism- and inflammation-related genes were determined by qRT-PCR and Western blot analysis, and the results are presented in Fig. 6(A–H). Compared with the HF group, hepatic TGF-β1, TGF-β2, TNF-α and uncoupling protein 2 (UCP-2) mRNA and protein expression level was significantly down-regulated in the different PSEs intervention groups (*P* < 0.05) (Fig. 6A–D). In addition, peroxisome proliferator-activated receptor-α (PPAR-α) and peroxisome proliferator-activated receptor-γ (PPAR-γ) expression was upregulated in the PSEL group and significantly down-regulated in the PSEH group (*P* < 0.05), respectively. (Fig. 6E,F).

PSEs intervention also had a positive effect on liver X receptor α (LXRα) expression. Specifically, LXRα expression was significantly higher in the PSEL group than in the HF group (*P* < 0.05) (Fig. 6G). Further, elongation of very-long-chain fatty acid enzyme 2 (ELOVL2) expression in the rat liver tissues exhibited a trend toward upregulation after the treatments with varying doses of PSEs, but the differences were not significant among the groups (Fig. 6H).

## Discussion

In present study, an NAFLD rat model was established using a diet enriched in saturated fat (lard) and cholesterol. The results showed that the blood TC and LDL-C concentrations were significantly increased at 7 and 12 weeks in the HF group compared with those in the NC group, indicating a lipid metabolic disturbance. Histopathological observation (HE and Oil Red O staining) also revealed severe hepatic steatosis in the HF group compared with the NC group. Previous studies have shown that the serum AST and ALT levels are mild to moderately elevate in patients with NAFLD^14^. The AST, ALT and ALP activities increased by 11, 31 and 24%, respectively, in the rats in the HF group compared with those in the NC group, indicating successful establishment of the NAFLD model.

Studies have shown that Ezetimibe, an LDL-C lowering agent that inhibits the absorption of cholesterol in the small intestine, has potential benefits in treating NAFLD^15^. This implied that the LDL-C level is closely related to the occurrence of NAFLD, and controlling this level is an important strategy for the prevention and progression of NAFLD. Plant sterols are a group of compounds present in plant-based foods, such as cereals and vegetable oils. Structurally, they resemble cholesterol but have much lower absorption rates, and reduce the serum cholesterol level by partially blocking cholesterol (TC, LDL-C) absorption in the digestive tract^16^. Our present study revealed that all doses of PSEs (0.05, 0.1, and 0.15 g/kg·BW, equivalent to 3, 6, and 9 g/d PSEs in human adults, respectively) reduced the serum LDL-C level. A low dose of PSEs (0.05 g/kg·BW) decreased this level by 18% by the 7^th^ week and by 14% by the end of the study. In addition, PSEs effectively reduced hepatic lipid (TG, TC, and FFA) accumulation. Histological examination further indicated that PSEs treatment significantly alleviated liver steatosis. Liver function improved, as indicated by the control of AST, ALT, and ALP activities to varying degrees. These results suggest that PSEs, as cholesterol absorption inhibitors, may have beneficial effects on the prevention and progression of NAFLD.

Notably, hyperuricemia is component of metabolic syndrome reflecting insulin resistance^17^, and is a common feature in NAFLD patients. The prevalence of NAFLD increases with increasing serum UA level^18^,^19^. Importantly, our renal function test results showed that the serum UA level increased by 51% in the HF group, while it was significantly lowered by PSEs treatment, similar to what was observed in the NC group at the end of the study.

In addition, oxidative stress and cytokine challenge are important in mediating the progression of NAFLD from steatosis to nonalcoholic steatohepatitis (NASH), fibrosis and cirrhosis. Fatty acid oxidation is an important source of reactive oxygen species (ROS) in fatty livers^20^. ROS attack polyunsaturated fatty acids and initiate lipid peroxidation within cells, resulting in the formation of aldehyde by-products, such as MDA. These molecules have the potential to diffuse from their sites of origin to reach distant intracellular and extracellular targets, thereby amplifying the effects of oxidative stress^21^. We further investigated the effects of PSEs on the oxidative status of the NAFLD rats by measuring the hepatic MDA level and SOD, CAT, and GPx activities. The results indicated that hepatic SOD activity was markedly increased and that the MDA concentration was reduced by PSEs at all doses. The antioxidant potential of PSEs may represent another mechanism for alleviating and improving liver function in NAFLD rats.

Importantly, XOD, an enzyme involved in the production of UA from purine nucleotides, is an important source of oxidative stress. Numerous recent studies have indicated that metabolic syndrome (MS), including NAFLD or steatohepatitis (NASH), is likely related to hyperuricemia^22^. Xu C *et al*.^23^ have also shown that NAFLD significantly increases the risk of incident hyperuricemia. Further, Nakatsu Y *et al*.^24^ have observed that febuxostat, an XOD inhibitor, exerts strong protective effects against NASH development induced by high-fat diet intake and that hyperuricemia. Elevated ALT, increased collagen deposition, inflammatory cytokine expression, and lipid peroxidation are normalized by febuxostat administration. These data suggest that XOD is a mediator of the relation between NAFLD and hyperuricemia and that it may serve as a novel therapeutic target for the two linked diseases. Our study showed that low and medium doses of PSEs could significantly decrease XOD activity. Considering that hepatic TG accumulation and IR are closely related to UA production^25^, the decreased hepatic TG, FBG and XOD activities after PSEs treatment may partly explain the negative effects of PSEs on the UA level.

The balance between cytokines/adipocytokines and pro- and anti-inflammatory activities plays a key role in the development of NAFLD. The pro-inflammatory cytokines TNF-α and IL-6 are critically involved in the various aspects of pathophysiology of human NAFLD, and are the major stimuli responsible for increased hepatic production of hs-CRP, fibrinogens and other acute-phase proteins. The level of hs-CRP, which is known to be a CVD risk factor, is increased in NAFLD patients, particularly in those with NASH^26^. Adiponectin is a potent TNF-α-neutralizing and anti-inflammatory adipocytokine. *In vitro* and experimental animal studies have demonstrated the importance of this mediator in counteracting inflammation and IR. The anti-inflammatory effects of adiponectin are mediated by the suppression of TNF-α synthesis and the induction of anti-inflammatory cytokines, such as IL-10 or IL-1 receptor antagonists. Zaitone SA *et al*.^27^ have observed that boswellic acids and pioglitazone improve insulin sensitivity and reduce the liver index, liver enzyme activity, and the serum TNF-α and IL-6 levels in rats fed a high-fat diet. Our study showed that PSEs significantly decreased the serum IL-6, IL-10, TGF-β and CRP levels and slightly increased the adiponectin and TNF-α levels, suggesting an anti-inflammatory potential of PSEs in NAFLD.

As mentioned above, steatosis can progress to NASH, which is characterized by hepatocyte injury due to inflammation and collagen deposition. Approximately 20% of patients with NASH develop cirrhosis, a condition in which hepatocytes are replaced by collagen tissue^28^,^29^. TGF-β has been shown to play an essential role in the development of liver fibrosis^30^. Provenzano A *et al*.^31^ have shown that severe necro-inflammation and fibrosis in a murine model of steatohepatitis are accompanied by increased expression of the pro-fibrogenic gene TGF-β. The decreased TGF-β level observed after administration of PSEs in the present study further demonstrated that PSEs could partly alleviate inflammation and fibrosis progression.

The above results demonstrate the protective effects of PSEs on NAFLD. To further explore the underlying molecular mechanisms, the hepatic mRNA and protein expression of some important genes involved in metabolism (PPAR-α, PPAR-γ, UCP2, ELOVL2, and LXRα) and inflammation (TGF-β1, TGF-β2, and TNF-α) was detected in our study. The treatments with different doses of PSEs obviously inhibited hepatic TGF-β1, TGF-β2, and TNF-α mRNA and protein expression, consistent with the trends in the serum protein levels.

TGF-β occurs in at least three isoforms, TGF-β1, TGF-β2 and TGF-β3, and it plays an essential role in the development of liver fibrosis^32^. During liver fibrosis, the TGF-β1 level is markedly increased in stellate cells, and TGF-β2 is also primarily expressed in Kupffer cells, followed by stellate and endothelial cells. A previous study has observed severe necro-inflammation and fibrosis in a murine model of steatohepatitis, accompanied by increased TGF-β expression^33^. Furthermore, TNF-α over-expression in an NAFLD ob/ob mouse model has also been reported, and treatment with anti-TNF antibodies (to inhibit TNF-α activity) has been shown to potentially improve NAFLD^34^. Therefore, the inhibition of TGF-β1, TGF-β2 and TNF-α expression by PSEs may partly explain its role in preventing the progression of liver steatosis to fibrosis and NASH.

PPARs are transcription factors that modulate the expression of genes involved in lipid metabolism, energy homeostasis and inflammation. Experimental evidence shows that PPAR-α is the master regulator of hepatic β-oxidation (mitochondrial and peroxisomal) and microsomal Ω-oxidation and that its expression is markedly decreased by high fat intake. Shi LJ *et al*.^35^ have suggested that the therapeutic effects of oxymatrine on hepatic steatosis in rats with high-fructose diet-induced fatty liver are partly due to up-regulation of the PPAR-α-mediated metabolic pathways. These observations prompted an attempt to treat NAFLD by targeting PPAR-α. In addition to activation of PPAR-α, partial PPAR-γ activation has beneficial effects, primarily mediated by increased adiponectin expression and decreased insulin resistance^36^. The results of our study showed that treatments with low and medium doses of PSEs (particularly a low dose) induced PPAR-α and PPAR-γ expression. Considering the above-mentioned roles of PPAR-α and PPAR-γ, they may be the underlying target genes that are induced in the PSE-mediated attenuation of the progression of NAFLD.

LXRα, a multi-functional gene, its expression was significantly upregulated by a low dose of PSEs. Kim DI *et al*.^37^ have shown that LXRα and protein arginine N-methyltransferase 3 (PRMT3) expressions are increased in cellular and mouse models of NAFLD and in humans. LXRα expression is correlated with the degree of hepatic fat deposition, hepatic inflammation and fibrosis in NAFLD patients^38^. While the activation of LXR results in a decrease in the blood glucose level in many diabetic animal models^39^. The effects of LXR activation on blood glucose are mediated by increases in insulin sensitivity and insulin secretion^40^,^41^. Therefore, the role of LXRα in prevention effect of PSE on NAFLD still need further investigation.

Moreover, hepatic long-chain fatty acid composition is a novel determinant in NASH development. ELOVL6 is a microsomal enzyme that regulates the elongation of C12–16 saturated and monounsaturated fatty acids. Its expression is positively correlated with the severity of hepatosteatosis and liver injury in NASH patients, and it could be a potential therapeutic target for the prevention and treatment of NASH^42^. In our study, we observed that different doses of PSEs induced the expression of ELOVL2. In most animals, ELOVL2 is essential for the conversion of dietaryα-linolenic acid (ALA) to docosahexaenoic acid (DHA) because only ELOVL2, and not ELOVL5, elongates docosapentaenoic acid (DPA, 22:5n-3) to 24:5n-3, the precursor of DHA. The exact role of ELOVL2 in the PSE-mediated prevention of NAFLD development also requires further investigation.

**Figure 7:**
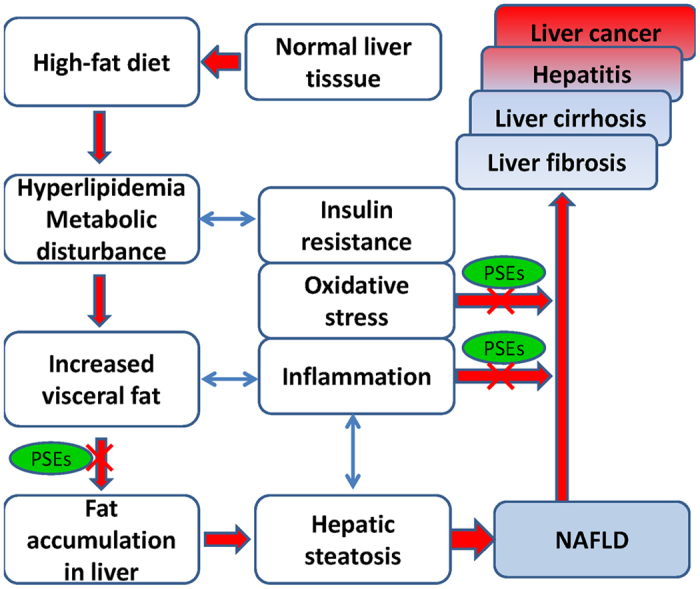
Targets through which PSEs play preventive roles against NAFLD.

In summary, treatment with PSEs improved liver histology, reduced hepatic total lipids, and decreased the serum ALT and AST levels in the rats with NAFLD. Low and medium doses of PSEs (equal to 3 or 6 g/d in humans) effectively prevented the occurrence and progression of NAFLD by lowering the blood LDL-C ameliorating oxidative stress and modulating key cytokines (as shown in Fig. 7). These benefits may be associated with adjustments in the hepatic mRNA and protein expression of TNF-α, TGF-β and certain metabolism-related genes (PPAR-α, PPAR-γ, UCP2, and LXRα). Further human studies are required to determine the potential of PSEs as therapeutic agents for the prevention and progression of NAFLD.

## Materials and Methods

### Chemical composition of phytosterol ester

Phytosterol esters (PSEs) (Vegapure^®^ 95FF: total PSEs and phytosterol content ≥97%; PSEs content ≥91%; and free phytosterol content ≤6%) had the following chemical composition: cholesterol ≤2%; brassicasterol ≤6%; campesterol 20.0–29.0%; stigmasterol 12.0–23.0%; β-sitosterol 42.0–55.0%; D5-oat sterol ≤4%; D7-oat sterol ≤2%; D7-stigmasterol ≤2%; and other ≤5%. The PSEs products were supplied by BASF China, Ltd.

### Experimental animals and treatment

Four-week-old male Sprague-Dawley rats (120 ± 10 g) were purchased from the Slack Laboratory Animal Co., Ltd. The experimental conditions and procedures were approved by the Shanghai Jiaotong University Institutional Animal Care and Use Committee and were consistent with the National Institutes of Health Guide for the Care and Use of Laboratory Animals.

At 5 weeks of age, the animals were randomized into the following five groups containing animals with similar mean body weights: a normal control group (NC, n = 7), high-fat diet group (HF, n = 12), low-dose PSE treatment group (PSEL, n = 12), medium-dose PSE treatment group (PSEM, n = 12) and high-dose PSE treatment group (PSEH, n = 12). The rats in the NC group were fed a standard diet, and those in the four other groups were fed a high-fat diet.

The SD rats in the three PSEs treatment groups were orally administered PSEs-fortified skimmed milk (1 mL/100 g·BW) once a day for 12 consecutive weeks. The rats in the three PSEs intervention groups were administered 0.05 g/100 g·BW, 0.10 g/100 g·BW or 0.15 g/100 g·BW of PSE, respectively, which equates to 3 g/d, 6 g/d and 9 g/d, respectively, in humans. As a control, skim milk was used in the NC and HF groups.

### Sample collection and analysis of serum biochemical parameters

For details, please see Supplementary Materials.

### Analysis of hepatic fat content and oxidation status

For details, please see Supplementary Materials.

### Cytokine assays

ELISA-based kits were used to determine the serum levels of TNF-α, TGF-β (BMS623 eBioscience), IL-10 (BMS629 eBioscience), IL-6 (BMS625 eBioscience), leptin (ELR-Leptin-1 RayBiotech) and adiponectin (Acrp30, R&D Systems) using a microplate reader (INFINITE M200PRO, Switzerland).

### High-sensitivity C-reactive protein (hs-CRP) measurement

The high sensitivity C-reactive protein (hs-CRP) level was assessed by enzyme immunoassay using the automated immunoturbidometric method (Cobas Integra 800, Roche diagnositics, Switzerland).

### Histological analysis

The livers were fixed in 10% buffered formalin, processed, and embedded in paraffin for hematoxylin-eosin (H&E) staining. Frozen samples prepared at the optimal cutting temperature were also stained with Oil Red O. The microscope slides were analyzed using ImageJ and Microsuite (Olympus Soft Imaging Solutions GmbH, Munster, Germany).

Histological scoring of hepatic steatosis, lobular inflammation, ballooning, and fibrosis was performed by pathologists who were blinded to the group assignments.

### RNA extraction and quantitative RT-PCR analysis

Total RNA was extracted from the liver tissues (50~100 mg of each sample) using a phenol-based method according to the manufacturer’s instructions (TRIzol^®^ Reagent, TakaRa, Dalian, China). cDNA synthesis was performed on the same day as total RNA extraction using SuperScript III reverse transcriptase (Invitrogen, USA). Reverse transcription was performed in the presence of random hexamers and oligo-dT.

### Quantitative RT-PCR

TGF-β, TNF-α, ELOVL2, LXRα, UCP2, PPARγ and PPARα mRNA expression levels were detected by quantitative RT-PCR using SYBR Premix Ex Taq™ (Code RR420A, Takara). Target gene expression (2^−ΔΔCt^) was normalized to endogenous β-actin expression. The primers used are listed in the Supplementary Data section (Table S1).

### Protein extraction and Western blotting

The rat livers were lysed in RIPA buffer (RIPA Lysis and Extraction Buffer, Thermo Scientific Pierce, USA; Cat# 89900), and the protein extracts were measured using a Bradford Assay Kit (Thermo Scientific Pierce, USA; Cat# 23200). Equal amounts of protein (50 μg) were heat denatured in 4× sample buffer (2% sodium dodecyl sulfate, 62.5 mM Tris (pH 6.8), 0.01% bromophenol blue, 1.43 mM mercaptoethanol, and 0.1% glycerol), separated on a 10% or 12% sodium dodecyl sulfate-polyacrylamide gel, and electroblotted onto a nitrocellulose membrane (Thermo Scientific Pierce, USA; Cat# 88025, Shanghai, China). The membranes were subsequently treated with the appropriate antibodies against the following proteins: TGF-β1 (1/1000) (Cat# ab179695, Abcam, Shanghai, China), TGF-β2 (1/500) (Cat# 167655, Abcam, Shanghai, China), TNF-α (1/1000) (Cat# ab66579, Abcam, Shanghai, China), UCP-2 (1/500) (Cat# ab67241, Abcam, Shanghai, China), PPAR-α (1/1500) (Cat# GTX101098, GeneTex, Shanghai, China), PPAR-γ (1/2000) (Cat# ab191407, Abcam, Shanghai, China), LXR-α (1/1000) (Cat# ab41902, Abcam, Shanghai, China), ELOVL-2 (1/5000) (Cat# ab176327, Abcam, Shanghai, China) and β-actin (1/10000) (Cat# a5441, Sigma-Aldrich, Shanghai, China).

### Statistical analysis

All parameters are expressed as the mean ± SD. The results were statistically analyzed by one-way ANOVA, followed by Tukey’s multiple comparison test. The criterion for significance was a *p* < 0.05. Analysis was performed using SPSS 19.0 software package (Analytical Software, USA).

## Additional Information

**How to cite this article:** Song, L. *et al*. Phytosterol esters attenuate hepatic steatosis in rats with non-alcoholic fatty liver disease rats fed a high-fat diet. *Sci. Rep.* **7**, 41604; doi: 10.1038/srep41604 (2017).

**Publisher's note:** Springer Nature remains neutral with regard to jurisdictional claims in published maps and institutional affiliations.

## Supplementary Material

Supplementary Methods and Figures

## Figures and Tables

**Table 1:** Effects of PSEs on rat liver function ( 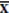 ± s).

Time	Indicator	NC	HF	PSE_L_	PSE_M_	PSE_H_
t_0_	AST (U/L)	270 ± 28	256 ± 47	272 ± 76	270 ± 39	283 ± 59
	ALT (U/L)	41 ± 3	43 ± 8	37 ± 4	39 ± 9	29 ± 9
	ALP (U/L)	443 ± 79	435 ± 60	407 ± 48	461 ± 81	469 ± 64
	DB (μmol/L)	0.5 ± 0.1	0.5 ± 0.1	0.4 ± 0.2	0.3 ± 0.2	0.4 ± 0.2
	TP (g/L)	59 ± 2	58 ± 2	59 ± 2	59 ± 2	59 ± 3
	ALB (g/L)	31 ± 1	31 ± 1	31 ± 1	31 ± 1	31 ± 2
t_7_	AST (U/L)	152 ± 28	178 ± 19	163 ± 38	147 ± 29^△^	161 ± 40
	ALT (U/L)	36 ± 7	52 ± 11^**^	52 ± 11^**^	53 ± 13^**^	57 ± 15^**^
	ALP (U/L)	184 ± 25	253 ± 34^**^	265 ± 54^**^	249 ± 59^**^	278 ± 62^**^
	DB (μmol/L)	0.4 ± 0.2	0.3 ± 0.1^**^	0.2 ± 0.1^**^	0.2 ± 0.1^**^	0.2 ± 0.1^**^
	TP (g/L)	74 ± 4	80 ± 4^**^	82 ± 3^**^	80 ± 5^**^	84 ± 4^**△^
	ALB (g/L)	33 ± 2	33 ± 2	34 ± 2	32 ± 2	34 ± 1^△^
t_12_	AST (U/L)	147 ± 24	163 ± 43	147 ± 25	160 ± 44	160 ± 38
	ALT (U/L)	48 ± 8	63 ± 12^*^	60 ± 14^*^	64 ± 13^*^	42 ± 13^ΔΔ^
	ALP (U/L)	116 ± 12	144 ± 16^*^	135 ± 28	120 ± 27^Δ^	133 ± 19
	DB (μmol/L)	0.7 ± 0.2	0.5 ± 0.2^*^	0.5 ± 0.1^*^	0.4 ± 0.2^**^	0.4 ± 0.2^**^
	TP (g/L)	58 ± 3	60 ± 4	61 ± 4	63 ± 2^**^	65 ± 4^**ΔΔ^
	ALB (g/L)	29 ± 1	27 ± 2	27 ± 2^*^	28 ± 1	26 ± 3^**^

Note: t_0_, t_7_, and t_12_ indicate data measured before the experiment, at the 7^th^ week and at the end of the experiment, respectively. The NC, HF, PSEL, PSEM and PSEH groups are described in Fig. 1. The data are expressed as the mean ± standard deviation. *P < 0.05 *vs.* NC, **P < 0.01 *vs.* NC, ^Δ^*P* < 0.05 *vs.* HF, ^ΔΔ^*P* < 0.01 *vs.* HF.

**Table 2:** Effects of PSEs on rat renal function ( 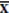 ± s).

Time	Indicator	NC	HF	PSE_L_	PSE_M_	PSE_H_
t_0_	Urea	8.4 ± 1.2	9.1 ± 1.5	8.9 ± 1.6	8.7 ± 2.4	9.2 ± 1.9
	CRE	20 ± 3	20 ± 3	20 ± 3	21 ± 4	19 ± 3
	UA	0.118 ± 0.006	0.116 ± 0.010	0.121 ± 0.015	0.121 ± 0.015	0.123 ± 0.013
t_7_	Urea	6.3 ± 0.9	6.0 ± 0.8	6.2 ± 0.9	5.7 ± 1.1	6.9 ± 0.9^△^
	CRE	30 ± 3	35 ± 3^**^	32 ± 4	31 ± 2^△^	33 ± 3^*^
	UA	0.078 ± 0.011	0.089 ± 0.012	0.087 ± 0.009	0.091 ± 0.019	0.087 ± 0.007
t_12_	Urea	5.8 ± 0.5	5.5 ± 0.3	5.6 ± 0.8	5.3 ± 0.6	5.4 ± 0.9
	CRE	26 ± 5	32 ± 4^**^	28 ± 5	27 ± 2^△^	29 ± 4
	UA	0.104 ± 0.022	0.157 ± 0.051^**^	0.096 ± 0.019^ΔΔ^	0.102 ± 0.025^ΔΔ^	0.101 ± 0.010^ΔΔ^

Note: t_0_, t_7_, and t_12_ indicate three time points, i.e. before the experiment, at the 7^th^ week of the experiment and at the 12^th^ week of the experiment, respectively. The data are expressed as the mean ± standard deviation. *P < 0.05 *vs.* NC, **P < 0.01 *vs.* NC, ^Δ^*P* < 0.05 *vs.* HF, ^ΔΔ^*P* < 0.01 *vs.* HF.
