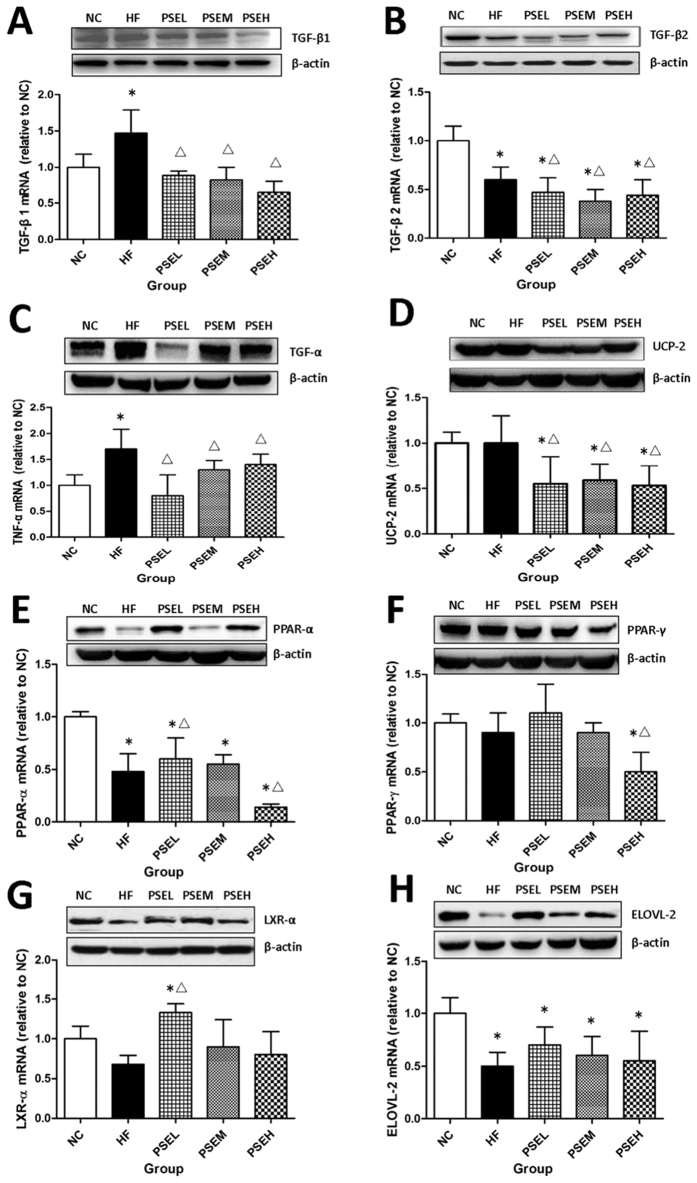# Corrigendum: Phytosterol esters attenuate hepatic steatosis in rats with non-alcoholic fatty liver disease rats fed a high-fat diet

**DOI:** 10.1038/srep46884

**Published:** 2017-08-24

**Authors:** Lihua Song, Dan Qu, Qing Zhang, Jing Jiang, Haiyue Zhou, Rui Jiang, Yating Li, Yao Zhang, Hongli Yan

Scientific Reports 7: Article number: 41604; 10.1038/srep41604 published online: 02 07 2017; updated: 08 24 2017.

This Article contains an error in the order of the Figures. Figures 3, 4 and 6 were published as Figures 4, 6 and 3 respectively. The correct Figures 3, 4 and 6 appear below as Figures 1, 2 and 3 respectively. The legends for the Figures are correct.

## Figures and Tables

**Figure 1:**
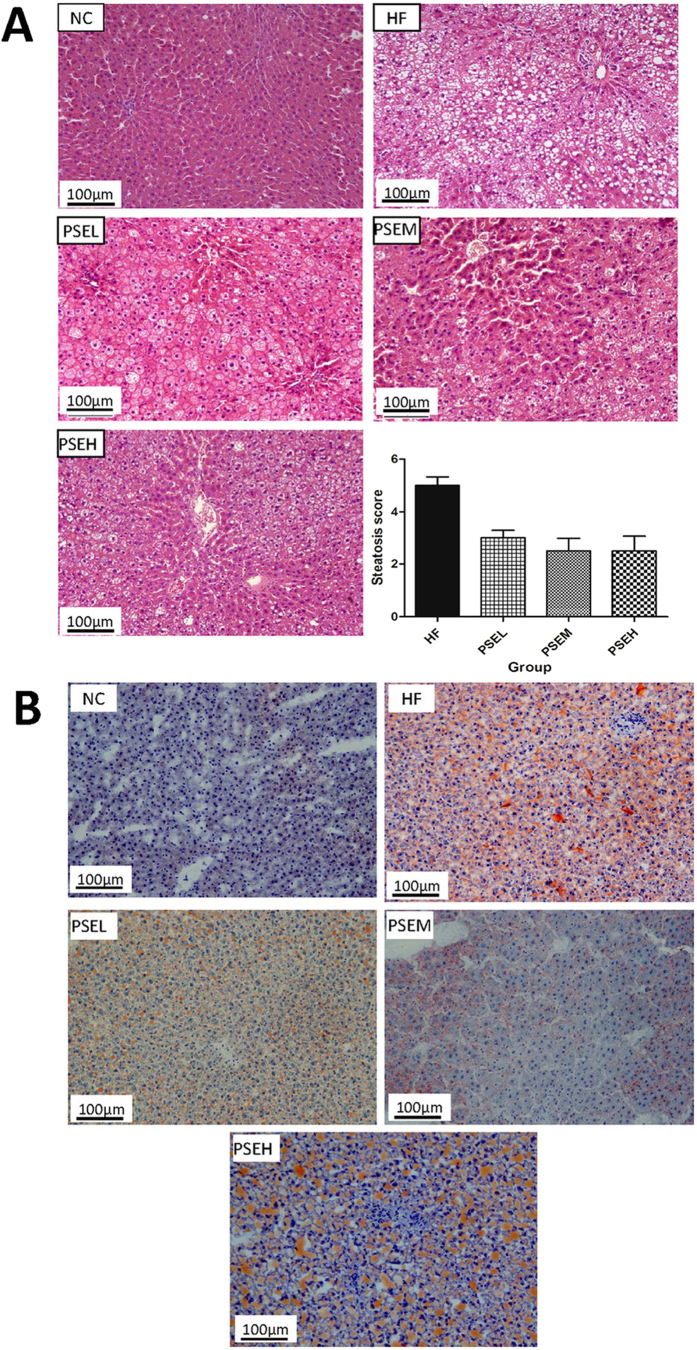


**Figure 2:**
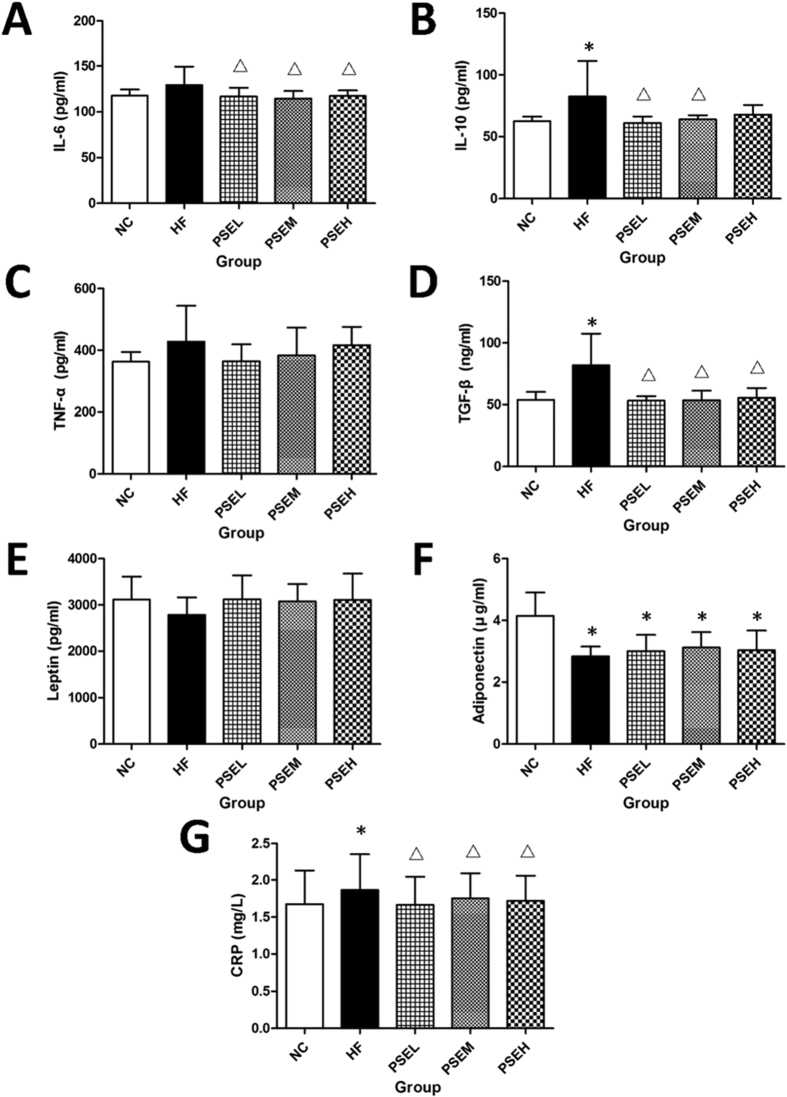


**Figure 3:**