# Supplementary material for: Phytosterol esters attenuate hepatic steatosis in rats with non-alcoholic fatty liver disease rats fed a high-fat diet
Source: Sci Rep. 2017 Feb 7;7:41604. doi: 10.1038/srep41604 (PMC5294417; doi:10.1038/srep41604)
Supplement: Supplementary Methods and Figures [file srep41604-s1.doc]

Phytosterol esters attenuate hepatic steatosis in rats with non-alcoholic fatty liver disease rats fed a high-fat diet

Lihua Song^1^, Dan Qu^3^, Qing Zhang^1^, Jingjiang^1^, Haiyue Zhou^1^, Rui Jiang^1^, Yating Li^1^, Yao Zhang^1^, Hongli Yan^2*^

**Supplenmentary Materials and methods**

The rats were housed in a 12-h light/dark cycle environment with free access to food and water. The temperature was maintained between 19 and 22°C. The relative air humidity was maintained at 70–80% and the rooms were extensively ventilated.

The composition of the standard and high-fat diets were noted as the following: standard diet: moisture content ≤10%, crude protein ≥20%, crude fat ≥4%, crude fiber ≤5%, crude ash ≤8%, minerals and amino acids 1.6-5.1%; high-fat diet: standard diet 54%, lard 18.4%, cholesterol 1%, choline salt 2%, saccharose 10%, casein 10%, maltodextrin 2.7% and other 1.9%.

**Sample collection and analysis of serum biochemical parameters**

At the beginning of the experiment, orbital blood samples were obtained and centrifuged to obtain the baseline data for each animal after fasting for 12 h. At the end of the experiment (12th week), the rats were weighed, anesthetized with chloral hydrate (0.2 ml/100 g·BW) and were euthanized by cervical dislocation according to recommendations for experimental animals. Blood samples were drawn from the abdominal aorta into test tubes, and the serum was prepared by solidification and low-speed centrifugation (3509 g, 10 min, 4°C). Pipetted aliquots of 500 μl were used immediately for the detection of serum lipids（TC, TG, LDL-C, HDL-C), liver function（AST, ALT, ALP, DB, TP, ALB), kidney function (urea, creatinine, UA) and fasting blood glucose (FBG) by direct measurement assays using an [automatic](javascript:void(0);) [biochemical](javascript:void(0);) [analyzer](javascript:void(0);) (Hitachi 7600-20, Japan). Aliquots of the remaining serum at 4°C were saved and stored at −80°C until analysis.

After laparotomy, the liver was removed from each rat, excised and fixed, using the same lobe of the liver and specimen size (1 cm × 0.6 cm × 0.2cm) for histological examination. The left liver samples were frozen immediately with liquid nitrogen and stored at −80°C until lipid analysis and RNA extraction.

**Analysis of hepatic fat and oxidation status**

TC, TG and free fatty acid (FFA) contents in the liver samples were determined using enzymatic assay kits, including tissue total cholesterol assay kit F002-2, tissue free fatty acid assay kit A042 and tissue triglyceride assay kit F001-2, respectively, from Nanjing Jiancheng Biological Engineering Institute (Nanjing, China). The antioxidant activities, including the SOD (A001-1), CAT (A007-1), GSH-Px (A005) and XOD (A002) activity as well as the lipid peroxidation product MDA (A003-1) in liver homogenates were determined by enzymatic methods using corresponding commercial kits (Nanjing Jiancheng Bioengineering Institute, Nanjing, China). Briefly, liver lipids (TG, TC, FFA) were extracted by homogenization in 1 mL of 2-propanol /100 mg liver and then shaken for 45 min. The samples were centrifuged at 3000 g for 10 min, and the supernatant was assayed for TG, FFA, and TC contents.

**RNA extraction and Quantitative RT-PCR analysis**

Total RNA was extracted from liver tissue (50~100 mg) using a phenol-based method according to the manufacturer’s instructions (TRIzol® Reagent, TakaRa, Dalian, China), and eluted in 10 µL of RNase-free water (Fisher Scientific, USA). The quantity and purity of the extracted RNA was determined by absorbance at 260 nm (A260) and at 280 nm (A280) as measured by a NanoDrop 2000 spectrophotometer (Thermo, USA). An A260/A280 ratio between 1.8 and 2.1 was considered as an indicator of high-quality RNA. The integrity of total RNA was verified by 1% agarose gel electrophoresis with ethidium bromide (10 μg/ml). The results were documented using the Molecular Imager system (Bio-Rad, USA). All total RNA samples demonstrated a 2:1 intensity ratio of sharp, clear 28S and 18S rRNA bands.

To prevent deterioration of RNA during storage, cDNA synthesis was performed on the same day as total RNA extraction using SuperScript III reverse transcriptase (Invitrogen, USA). Reverse transcription was accomplished in the presence of random hexamers and oligo-dT. Equal volumes (1 µl) of the resulting cDNA served as templates for the subsequent PCR reactions.

**Quantitative RT-PCR**

TGF-β, TNF-α, ELOVL2, LXRα, UCP2, PPARγ and PPARα mRNA expression levels were detected by quantitative RT-PCR using SYBR Premix Ex Taq™ (Code RR420A, Takara). PCR amplification reactions were performed in 10 μl containing 1 μl of template cDNA. Quantitative real-time PCR was performed using an Eppendorf realplex 4 PCR system (Eppendorf, Germany). Ct values were used to calculate the RNA expression levels. The amount of target gene expression (2^-ΔΔCt^) was normalized to the endogenous β-actin. The primers used were list in supplenmentary data.

Specific primers were designed for each gene of interest followed by standard PCR reaction chemistry with the addition of the fluorescent DNA-binding dye, SYBR Green I (TAKARA, RR420A, Dalian, China) (Table S1).


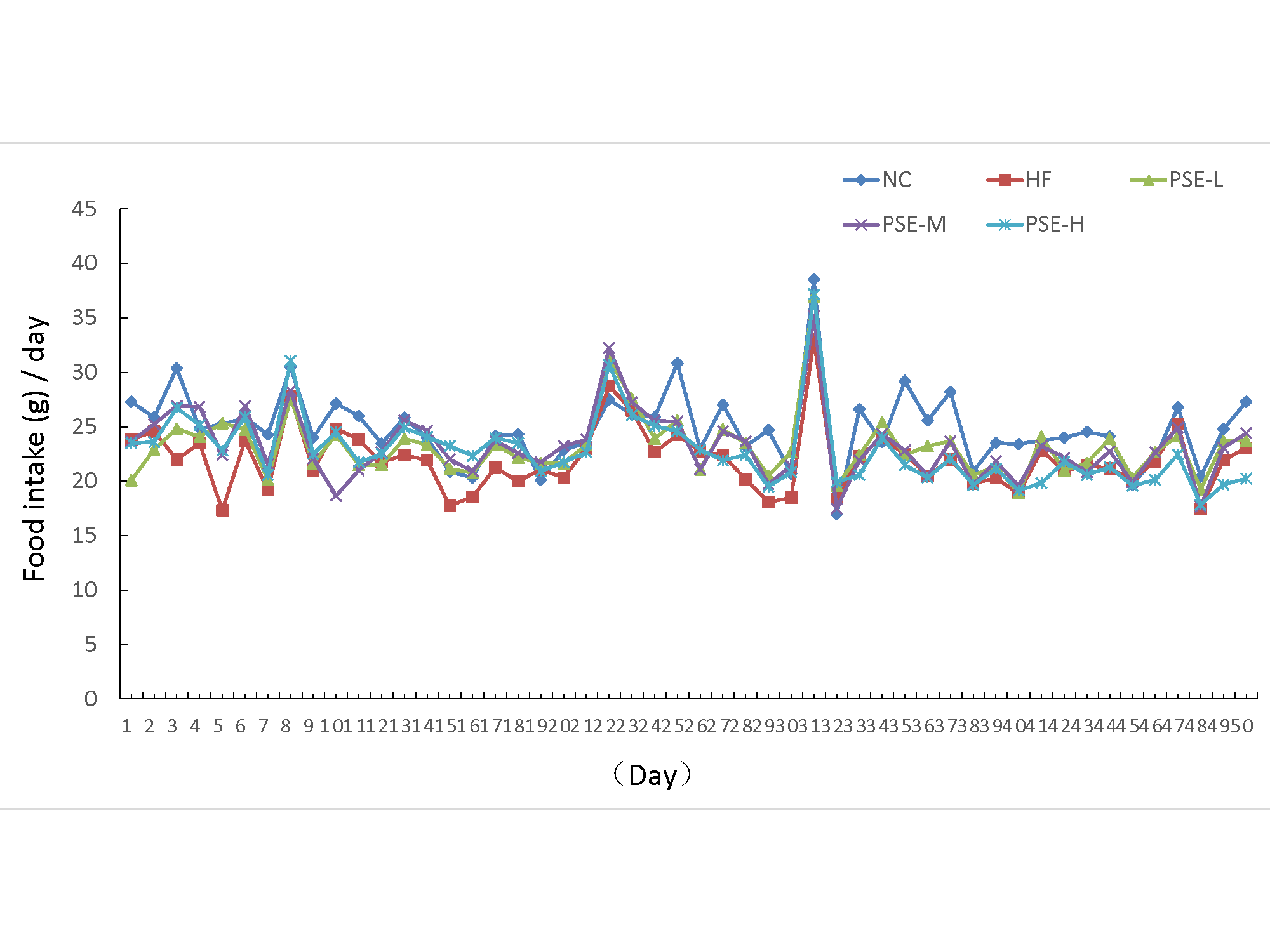


Fig.S1 Food intake in each group.

**Table S**1 Primer sequence of genes

| Gene |  | Primer sequences（5'-3'） |
| --- | --- | --- |
| UCP2 | Fw | GGTAAAGGTCCGCTTCCAGG |
|  | Rv | GCAAGGGAGGTCGTCTGTCA |
| TNF-α | Fw | ACTGAACTTCGGGGTGATTG |
|  | Rv | GCTTGGTGGTTTGCTACGAC |
| ELOVL2 | Fw | TTTGGCTGTCTCATCTTCCA |
|  | Rv | GGGAAACCATTCTTCACTTC |
| LXRα | Fw | TCAGCATCTTCTCTGCAGACCGG |
|  | Rv | TCATTAGCATCCGTGGGAACA |
| TGF-β1 | Fw | GAGAGCCCTGGATACCAACTACTG |
|  | Rv | GTGTGTCCAGGCTCCAAATGTAG |
| TGF-β2 | Fw | GCGAGCGAAGCGACGAGGAG |
|  | Rv | TGGGCGGGATGGCATCAAGGTA |
| PPAR-α | Fw | CACCCTCTCTCCAGCTTCCA |
|  | Rv | GCCTTGTCCCCACATATTCG |
| PPAR-γ | Fw | ATGGAGCCTAAGTTTGAGTTTGCT |
|  | Rv | GGATGTCCTCGATGGGCTTCA |
| β-actin | Fw | AGAGGGAAATCGTGCGTGAC |
|  | Rv | CGATAGTGATGACCTGACCGT |

The thermal profile of the QPCR procedure repeated for 40 cycles was as follows: 1) 95°C for 30 s; and 2) 5 s denaturation at 95°C, 20 s annealing at 60°C. Melting curves were used to validate product specificity. All samples were amplified in triplicate from the same total RNA preparation and the mean value was used for further analysis.

**Table S2 The effects of different PSE doses on weight of rats**（x ± s）

| Group | n | 0w | 1w | 2w | 3w | 4w | 5w | 6w |
| --- | --- | --- | --- | --- | --- | --- | --- | --- |
| NC | 7 | 148±8 | 191±15 | 246±16 | 284±15 | 336±20 | 375±19 | 402±23 |
| HF | 12 | 149±6 | 193±8 | 246±13 | 295±17 | 342±26 | 381±22 | 414±25 |
| PSEL | 12 | 153±8 | 196±10 | 257±16 | 304±19 | 336±34 | 383±37 | 418±40 |
| PSEM | 12 | 153±8 | 195±10 | 263±20 | 313±23 | 358±30 | 405±34 | 440±36 |
| PSEH | 12 | 151±6 | 189±10 | 248±15 | 293±22 | 330±21 | 380±27 | 411±28 |

| Group | n | 7w | 8w | 9w | 10w | 11w | 12w |
| --- | --- | --- | --- | --- | --- | --- | --- |
| NC | 7 | 434±29 | 451±30 | 476±33 | 494±35 | 509±37 | 526±41 |
| HF | 12 | 446±31 | 471±31 | 493±34 | 518±37 | 532±37 | 544±41 |
| PSEL | 12 | 458±45 | 485±47 | 509±52 | 535±50 | 553±53 | 565±59 |
| PSEM | 12 | 475±31 | 499±35 | 521±36 | 548±41 | 567±47 | 579±53 |
| PSEH | 12 | 449±28 | 471±31 | 490±36 | 519±37 | 528±36 | 500±80 |
